# Exploring novel kinetics of automated H_2_O_2_ nebulization: a breakthrough in SARS-CoV-2 elimination

**DOI:** 10.1128/spectrum.00842-25

**Published:** 2026-04-03

**Authors:** Jennifer Solano-Parada, José Antonio Sánchez-Martínez, Beatriz Carolina Gómez-Hernández, Margarita Barriga, Pilar García-Velasco, Alberto Fenández, Alberto Cornet-Gómez, Antonio Osuna Carrillo de Albornoz, Rene Fabregas, Concepción Morales-García

**Affiliations:** 1 Department of Parasitology, CTS183 Group, University of Granada, Institute of Biotechnology 16741, Granada, Spain; 2 Department of Pneumology, University Hospital Virgen de Las Nieves, Granada, Spain; 3 Biosanitary Research Institute of Granada-Ibs 616465 , Granada, Spain; 4 Department of Applied Mathematics and Modeling Nature (MNat) Research Unit, Faculty of Sciences, University of Granada 16741, Granada, Spain; CEA-Genoscope, Evry, France

**Keywords:** SARS-CoV-2, hydrogen peroxide nebulization, decontamination, kinetic modeling, RT- qPCR, infectivity assay, COVID-19 patient rooms, ductFIT, photocatalysis, machine learning

## Abstract

Although hydrogen peroxide (H_2_O_2_) nebulization has shown promise for reducing SARS-CoV-2 loads in healthcare settings, its precise kinetics and real-world efficacy remain incompletely understood. To address this, we conducted a prospective environmental study in COVID-19 patient rooms, performing a tripartite assessment of automated H_2_O_2_ nebulization. We collected air and surface samples before and after treatment, quantifying viral RNA (RT-qPCR) and antigens (indirect ELISA), and evaluating infectivity in Vero E6 cell cultures. We then applied a piecewise exponential model to characterize the virucidal kinetics, successfully capturing both initial delay and subsequent decay phases. The treatment proved highly effective, with reductions in environmental SARS-CoV-2 RNA load expressed in log_10_ units and uncertainty quantified by 95% confidence intervals. Results revealed a marked decrease in RT-qPCR positivity rates in air samples, from 55.6% to 22.2%, along with higher cycle threshold values indicative of lower viral loads, and substantially reduced cytopathic effects. Crucially, this suggests that the residual RNA detected corresponded to non-viable virus. Our findings demonstrate the non-linear dynamics of H_2_O_2_-mediated decontamination and highlight the influence of environmental variables. By integrating molecular, biological, and mathematical analyses, this study provides a robust framework for optimizing disinfection protocols. Future multi-centre studies should validate these models to enhance preparedness against emerging viral threats in diverse clinical environments.

**IMPORTANCE:**

This study provides a critical, multi-faceted validation of automated hydrogen peroxide (H_2_O_2_) nebulization using ductFIT photocatalysis systems as a tool for eliminating SARS-CoV-2 in hospital environments. Our work moves beyond simple pre- and post-treatment comparisons by integrating molecular detection, direct infectivity assays, and sophisticated kinetic modeling. This tripartite approach allows us to characterize the non-linear dynamics of viral decay and provides compelling evidence that residual viral RNA detected after decontamination corresponds to non-viable virus. These findings offer a robust, data-driven framework for optimizing and personalizing disinfection protocols to enhance patient and healthcare worker safety. Furthermore, by demonstrating the novel application of machine learning to augment sparse experimental data, we introduce a powerful method for improving the predictive accuracy of decontamination models. This research provides actionable insights for mitigating nosocomial transmission and strengthening preparedness against future airborne pathogens.

## INTRODUCTION

Ongoing COVID-19 outbreaks in healthcare facilities underscore the critical need for comprehensive disinfection protocols to mitigate viral transmission. While respiratory droplets are the primary transmission route, studies by Wölfel et al. (1) and others confirm that contaminated surfaces and airborne particles also contribute significantly to nosocomial spread (2). The persistence of SARS-CoV-2 on hospital surfaces makes them potential reservoirs for infection, posing a continuous risk to patients and healthcare staff. The virus’s lipid envelope, essential for its infectivity, is notably susceptible to oxidizing agents and surfactants (3). Rigorous environmental disinfection is, therefore, an essential component of a multilayered infection prevention strategy, complementing standard droplet and contact precautions (4).

Hydrogen peroxide (H_2_O_2_) nebulization has emerged as a compelling decontamination methodology for healthcare environments (5–7). Its appeal lies in its broad-spectrum antimicrobial activity, proven efficacy against enveloped viruses, and its capacity to decontaminate both surfaces and air in enclosed settings (8). Biochemically, H_2_O_2_ acts by generating hydroxyl free radicals (OH•), highly reactive oxidants that damage essential microbial components such as lipids, proteins, and nucleic acids (9). Indeed, vaporized hydrogen peroxide is well-documented to inactivate a wide array of pathogens in hospitals, including in difficult-to-reach areas (10). While previous work has substantiated the virucidal properties of H_2_O_2_ against various coronaviruses (2, 11–13), a rigorous evaluation of its efficacy against SARS-CoV-2 under real-world hospital conditions is needed to refine disinfection protocols (14, 15).

Reverse transcription quantitative PCR (RT-qPCR) is a primary tool for environmental SARS-CoV-2 surveillance (16). However, it has a key limitation: while highly sensitive for viral RNA, RT-qPCR cannot distinguish between infectious virions and non-viable viral fragments (17–19). Consequently, RT-qPCR data alone cannot confirm the risk of infection from environmental contamination or validate that a decontamination protocol has successfully eliminated infectious virus. To address this deficiency, viral culture on susceptible cell lines, such as Vero E6, is essential for directly assessing viral infectivity (1). Combining these methods provides a powerful, nuanced approach (20). RT-qPCR measures the quantity of viral RNA present, whereas viral culture directly assesses the infectivity of the virus itself. This integrated strategy, which is critical for accurately evaluating decontamination efficacy, ensures that observed reductions in RNA levels correlate with a genuine decrease in transmission risk (21). While diagnostic tools like ELISA are vital for patient care and epidemiological studies (22–26), the RT-qPCR and culture pairing is uniquely suited for rigorous environmental risk assessment.

In this study, we hypothesized that an automated H_2_O_2_ nebulization system could markedly reduce SARS-CoV-2 loads in hospital environments. Our primary objective was to move beyond simple pre- and post-treatment comparisons to characterize the *non-linear kinetics* of viral inactivation. To achieve this, we designed a tripartite assessment strategy that systematically integrates three pillars of analysis: (i) quantification of viral RNA in air and surface samples via RT-qPCR to measure total viral load, (ii) infectivity assays in Vero E6 cells to determine the viability of the detected virus, and (iii) the application of piecewise exponential models to capture the complex temporal dynamics of the decontamination process. By elucidating key kinetic parameters, such as delay phases and decay rates, this work provides a rigorous, data-driven framework for optimizing H_2_O_2_ disinfection protocols and offers robust, evidence-based insights into mitigating the risk of SARS-CoV-2 transmission in clinical settings.

## RESULTS

### Efficacy of nebulization on viral RNA reduction

To determine the total environmental viral RNA load, we performed direct RT-qPCR analysis on aliquots taken from each air and surface sample immediately after collection. A thorough environmental sampling study was conducted in 18 confirmed COVID-19 patient rooms, yielding a total of 72 samples (36 from air, 36 from surfaces) collected both before (b_t_) and after (a_t_) a standardized H_2_O_2_ nebulization protocol. We performed direct RT-qPCR analysis on these environmental samples to quantify viral RNA targeting the SARS-CoV-2 N-gene, using the Genestore Detection Expert 1S kit. In accordance with the manufacturer’s protocol, samples exhibiting a cycle threshold (Ct) value below 38 were classified as positive, while those with no amplification were assigned a Ct of 0. The consistently strong amplification of internal controls (mean Ct = 26.2 ± 2.5) affirmed the integrity of the RNA extraction and the minimal presence of PCR inhibitors, ensuring the reliability of our results.

Prior to decontamination, viral RNA was widespread. Of the 36 air samples, 20 tested positive (55.6%; 95% CI: 38.1%–72.1%), with Ct values ranging from 23.48 to 36.39. Concurrently, 16 of the 36 surface swabs (44.4%; 95% CI: 27.9%–62.0%) were positive, with Ct values between 24.79 and 33.46 (complete data are available in Table S3). Following H_2_O_2_ nebulization, we observed a statistically significant reduction in viral RNA detection across both sample types.

The number of positive air samples decreased from 20 to 8 (a reduction to 22.2%; 95% CI: 10.1%–40.1%; *P* = 0.014, Fisher’s exact test). Similarly, positive surface swabs fell from 16 to 5 (a reduction to 13.9%; 95% CI: 4.7%–30.0%; *P* = 0.028, Fisher’s exact test). For the subset of samples that remained positive post-treatment, we observed a marked and statistically significant increase in Ct values (mean difference = 4.8, 95% CI for the difference: 2.5–7.1; paired *t*-test, *P* < 0.001), indicating a substantial reduction in viral RNA load. For example, 58.3% of all previously positive samples became undetectable after treatment, while the Ct values of the remaining positive samples all increased to above 30. These findings, which align with the decontamination efficacy documented by Doremalen et al. (2) and Chin et al. (3), provide strong molecular evidence of the system’s capacity to reduce environmental viral contamination. Assuming a conventional RT-qPCR efficiency (3.32 cycles per log_10_), the observed paired ∆Ct maps to a reduction in log_10_ units via ∆ log10 = ∆*Ct/*3.32.

The distribution of Ct values pre-treatment exhibited bimodal characteristics, with 45% of positive samples showing Ct <30 (indicative of high viral loads) and 55% displaying Ct 30–38 (moderate to low viral loads). Post-treatment, all residual positive samples shifted to Ct >30, with 62.5% exceeding Ct 35, suggesting minimal residual viral RNA.

### Viral propagation and ELISA-based detection

To assess the presence of infectious SARS-CoV-2 and the efficacy of the decontamination protocol, environmental samples were inoculated onto susceptible Vero E6 cell cultures. Viral replication was evaluated by microscopic observation of cytopathic effects (CPE), such as cell rounding and detachment. Before decontamination (b_t_), 25 out of the 72 cultures (34.7%; 95% CI: 24.2%–46.7%) exhibited mild to moderate CPE, which strongly correlated with positive RT-qPCR results. Stratification by Ct value revealed that 92% (23/25) of CPE-positive cultures originated from samples with Ct <30, while only 8% (2/25) derived from Ct 30–35 samples, and none from Ct >35 samples. This stratification validates the established threshold of Ct ∼30 for predicting infectious virus presence (1, 20). Notably, 58.3% of samples becoming undetectable post-treatment corresponded predominantly to those with initial Ct values >28, while samples with Ct <28 pre-treatment exhibited Ct shifts but remained detectable, underscoring the relationship between initial viral load and decontamination kinetics explored further in Modelling decontamination H_2_O_2_ nebulisation kinetics. To quantify the viral antigen load in the culture supernatants, we employed an indirect ELISA targeting the SARS-CoV-2 S2 subunit.

**Fig 1:**
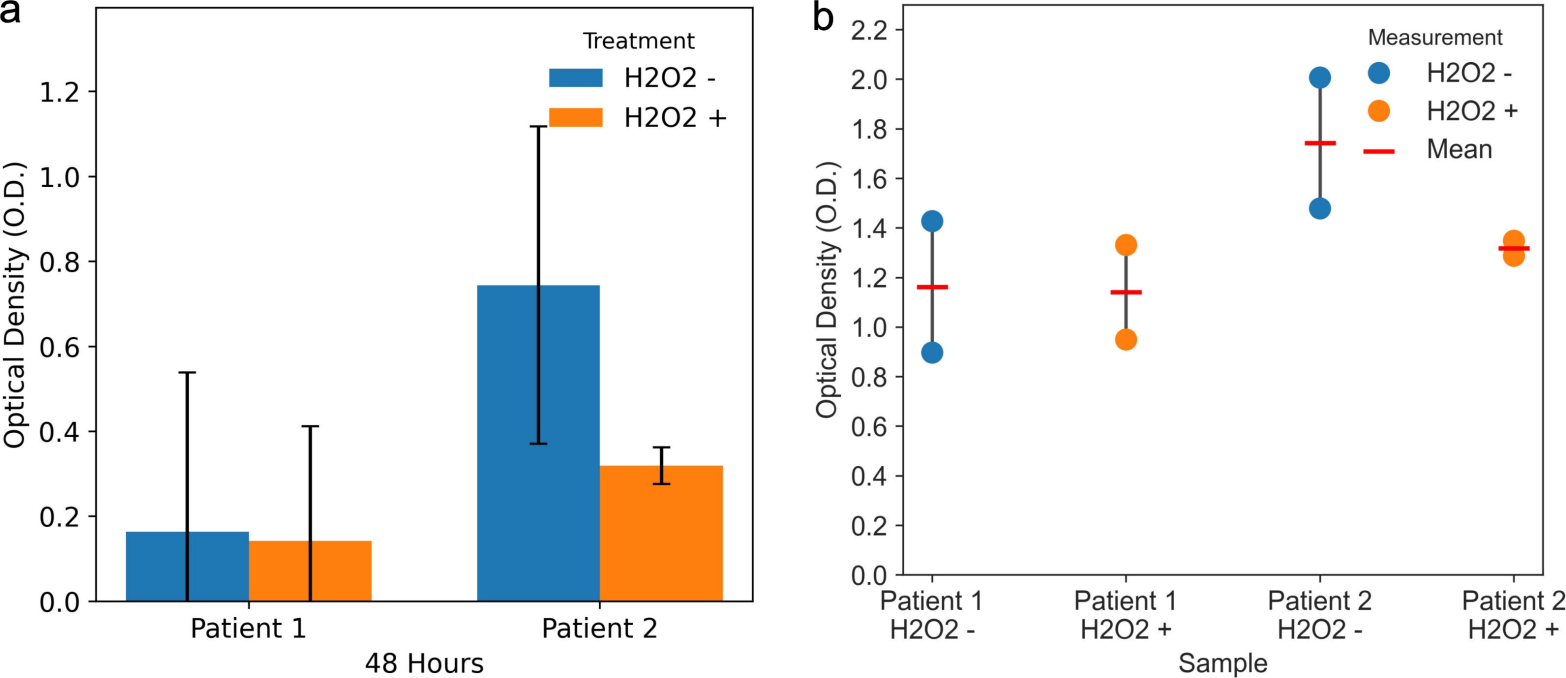
H_2_O_2_ nebulization effectively reduces SARS-CoV-2 antigen levels in culture supernatants derived from environmental samples. (**a**) Mean optical density (O.D.) values from air samples of two patients, collected before (H_2_O_2_ –) and after (H_2_O_2_ +) decontamination. Bars represent the mean of two biological replicates, and error bars depict the standard deviation. These data are derived from the measurements shown in Table S1. (**b**) Paired dot plot providing a granular view of the same data. Individual O.D. values for each replicate are shown as circles, connected by a gray line to visualize intra-assay variability. The corresponding mean for each sample is marked with a red horizontal line.

The pre-decontamination samples revealed optical density (OD) values significantly above the established positivity threshold (*P* < 0.05), indicating a substantial presence of replicating virus. Following decontamination (at), a statistically significant reduction in infectivity was observed, with only 4 of the 72 cultures (5.6%; 95% CI: 1.5%–13.5%) showing minimal CPE. This corresponded to a marked decrease in viral antigen levels as measured by ELISA. For instance, in the air sample from Patient 2, which had a high initial viral load, the mean OD value was reduced by more than half, from 0.7443 ± 0.3734 pre-treatment to 0.3196 ± 0.0428 post-treatment. Similarly, Patient 1 exhibited a reduction from 0.1639 ± 0.3750 to 0.1420 ± 0.2698, representing a 13.4% decrease. Across all patients with detectable pre-treatment antigen levels (*n* = 6), the mean OD reduction was 58.2% ± 18.7%, with individual reductions ranging from 13.4% to 91.2%, reflecting variability in initial contamination levels and environmental factors discussed in the kinetic analysis ( Modelling decontamination H_2_O_2_ nebulisation kinetics). Across all samples, the post-decontamination OD values fell below the positivity threshold (Fig. 1a). Paired replicate measurements demonstrated high intra-assay reproducibility (Fig. 1b), with individual OD values showing consistent reduction patterns across biological replicates. This dramatic 84% reduction in CPE-positive cultures, coupled with the significant decrease in ELISA-detected viral antigens, underscores the robust efficacy of the H_2_O_2_ nebulization protocol in eliminating infectious SARS-CoV-2 from the environment.

### Modeling decontamination H_2_O_2_ nebulization kinetics

To characterize H_2_O_2_ nebulization kinetics, we employed a piecewise exponential model to account for non-linear decontamination processes (*formalism in the Supplemental material*). This framework partitions decontamination into distinct phases, resolving an initial stabilization phase—reflecting aerosol settling or nebulizer initiation—from a subsequent log-linear decay phase with constant rate, indicative of effective H_2_O_2_ action. Using environmental SARS-CoV-2 RNA data from Patient 2 (Table S1), we parameterized observed RNA-load reductions following H_2_O_2_ nebulization.

**Fig 2:**
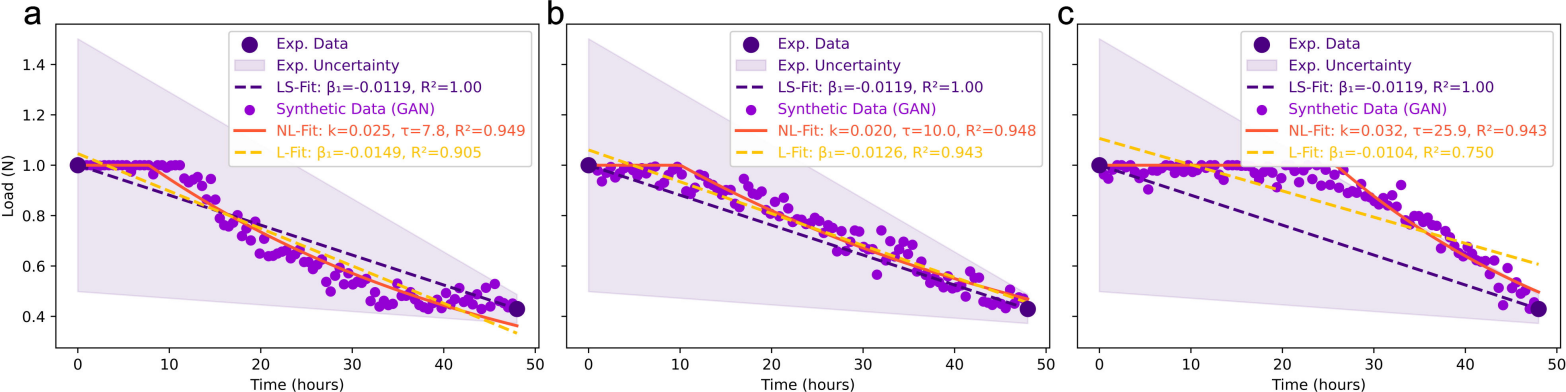
Comparison of three kinetic regimes. Rapid-onset followed by log-linear decay (**a**), uniform log-linear decay (**b**), and delayed-onset followed by steep log-linear decay (**c**) applied to Patient 2 data (Table S1). Purple markers denote RT-qPCR measurements of SARS-CoV-2 RNA, and lavender bands indicate experimental uncertainty; solid curves are non-linear least-squares (NL-Fit) piecewise models, and dashed lines are linear least-squares (LS-Fit). Orange markers are GAN-synthetic points used for visual interpolation only. These synthetic points were not used for model fitting, hypothesis testing, or AICc-based model selection. Panel legends report the corresponding linear slopes as *β*1 (LS-Fit) for the experimental data and *β*1 (L-Fit) for the GAN-synthetic points.

Figure 2 contrasts three kinetic regimes—*rapid-onset followed by log-linear decay* (Fig. 2a), *uniform log-linear decay* (Fig. 2b), and *delayed-onset followed by steep log-linear decay* (Fig. 2c)—fitted to RT-qPCR measurements (purple markers) with experimental uncertainty (lavender bands). For each regime, we estimated the virucidal rate constant (*k*, h^−1^) and the delay time (*τ*, h) by non-linear least squares (NL-Fit; solid lines); linear least-squares fits (LS-Fit; dashed lines) are shown for descriptive comparison. GAN-generated synthetic points (orange) provide visual interpolation across sampling gaps and are not used for inferential fitting.

In the rapid-onset regime (Fig. 2a), the piecewise model yielded *k* ≈ 0.025 h^−1^ and *τ* ≈ 7.8 h with descriptive *R*^2^ ≈ 0.95 (LS-Fit *R*^2^ ≈ 0.91). The uniform regime (Fig. 2b) gave *k* ≈ 0.020 h^−1^ and *τ* ≈ 10.0 h (*R*^2^ ≈ 0.95; LS-Fit *R*^2^ ≈ 0.94). The delayed-onset regime (Fig. 2c) exhibited a longer delay (*τ* ≈ 25.9 h) but a higher rate (*k* ≈ 0.032 h^−1^), with *R*^2^ ≈ 0.94 (LS-Fit *R*^2^ ≈ 0.75). Because *R*^2^ is not appropriate for non-nested, non-linear model comparison, we base model selection on AICc (small-sample correction; see Supplemental material). AICc weights favor the delayed-onset regime (*w*_rapid_ = 0.03, *w*_uniform_ = 0.21, *w*_delayed_ = 0.76).

While *R*^2^ is inadequate for formal model selection among non-nested alternatives, the descriptive fit quality improvements when incorporating delay parameters—*R*^2^ increases of 0.04 (Fig. 2a), 0.01 (Fig. 2b), and 0.19 (Fig. 2c) relative to linear fits—indicate that temporal lag phases constitute measurable features of H_2_O_2_ decontamination kinetics rather than experimental noise. The substantial ∆*R*^2^ = 0.19 in the delayed-onset regime (Fig. 2c) suggests that this scenario’s biphasic character is particularly sensitive to environmental heterogeneities, corroborating the AICc-based model selection favoring this regime.

Differences in fitted rates translate into distinct characteristic times. Within the exponential phase, the half-life is *t*_1_*/*_2_ = ln 2*/k*, yielding *t*_1_*/*_2_ ≈ 27.7 h (rapid-onset), 34.7 h (uniform), and 21.7 h (delayed-onset) before accounting for the respective delays. Accordingly, the total time to halve the load after initiation of nebulization is approximately *τ + t*_1_*/*_2_ for each regime. Within this piecewise framework, the delay parameter *τ* is interpreted mechanistically as reflecting a distribution of resistance times across sub-populations rather than a fixed lag, consistent with heterogeneity in H_2_O_2_ penetration and micro-environmental shielding (see Supplemental material).

Figure 2 further delineates decay patterns by contrasting the experimental linear least-squares slope and the GAN-assisted linear slope reported in the panel legends. Specifically, we use *β*_1_ (LS-Fit) = −0.0119 N h^−1^ for the experimental least-squares fit to the measured data, and *β*1 (L-Fit) for the linear fit computed on GAN-synthetic points; both slopes are expressed in N h^−1^. Accordingly, the rapid-onset scenario (Fig. 2a) shows *β*_1_ (L-Fit) = −0.0149 N h^−1^, the uniform scenario (Fig. 2b) *β*_1_ (L-Fit) = −0.0126 N h^−1^, and the delayed-onset scenario (Fig. 2c) *β*_1_ (L-Fit) = −0.0104 N h^−1^. Accordingly, rather than extrapolating time to 99% decontamination from linear slopes, we characterize clearance using model-consistent quantities—the onset delay *τ* and the exponential half-life *t*_1_*/*_2_ = ln 2*/k*. These values confirm that, within the observed window, the delayed-onset regime exhibits the shallowest decline, whereas the rapid-onset regime exhibits the steepest.

These kinetic distinctions have direct operational implications. The rapid-onset regime, characterized by brief delay (*τ* ≈ 8 h) and moderate decay rate, achieves effective decontamination within approximately 8–12 h in well-ventilated environments. Conversely, the delayed-onset regime requires substantially longer total nebulization times (>30 h) due to the extended quiescence period (*τ* ≈ 26 h), underscoring the necessity for elevated H_2_O_2_ concentrations or improved ventilation in enclosed high-humidity settings. These findings highlight the critical role of real-time environmental monitoring for adaptive protocol optimization.

## MATERIALS AND METHODS

### Recombinant protein production

The S1 (2.1 kb) and S2 (2.3 kb) genes were PCR-amplified from plasmid VG40589-CY (Sino Biological Beijing, China) using primers (Table 1) flanked by BamHI/SalI restriction sites, see Table S1.

**TABLE 1:** Primer sequences employed for the amplification of the S1 and S2 regions^*a*^

Primer	Sequence
S1_Forward	GGTGGTGGATCC TTTGTGTTCCTGGT
S1_Reverse	GGTGGTGTCGACTTACCTTGCCCTCCTTGG
S2_Forward	GGTGGTGGATCCTCTGTGG -CAAGCCAG
S2_Reverse	GGTGGTGTC GACTTAGGTGTAGTGCAGTTTC

^*a*^ These oligonucleotides were meticulously designed to ensure specificity and optimal performance during PCR amplification.

Digested products (Table 1) were ligated into pET28a (Novagen) and transformed into *E. coli* Rosetta(DE3) via heat shock (42°C, 45 s). Protein expression was induced with 1 mM isopropyl *β-d-1-thiogalactopyranoside (IPTG) for 4 h at 37°C in LB medium containing 50 μg/mL kanamycin and 35 μg*/mL chloramphenicol.

Cells were lysed in buffer containing 50 mM Tris-HCl (pH 8.0), 1 mg/mL lysozyme, 0.1% Triton X-100, and protease inhibitors (Roche). Inclusion bodies were solubilized in 8 M urea (pH 7.4) and purified by immobilized metal affinity chromatography (IMAC) using HisPrep FF 16/10 columns (Cytiva) on an ÄKTA Purifier system (GE Healthcare). Eluted fractions were analyzed by 12% SDS-PAGE and Western blotting with anti-His Tag monoclonal antibody (1:5,000; Sigma-Aldrich). Successful expression and purification of the recombinant S2 subunit was confirmed by SDS-PAGE and Western blot analysis, as detailed in Fig. S1.

### Expression of S1 and S2 proteins in *E. coli* cultures

The *Rossetta* strain of E. coli was transformed with the expression vectors pET28a-S1 and pET28a-S2 containing genes amplified with specific primers (Table 1) for the S2 protein. Bacterial cultures were grown in Luria-Bertani (LB) broth 10 g/L tryptone, 10 g/L NaCl, and 5 g/L yeast extract, supplemented with 50 pg/mL kanamycin and 35 pg/mL chloramphenicol.

Recombinant protein expression was induced with 1 mM IPTG for 4 h at 37°C. Bacterial cells were collected by centrifugation and stored at −20°C.

The cell pellets obtained from centrifugation were resuspended in lysis buffer (50 mM Tris, pH 8, 0.5 M NaCl, 10 mM EDTA, 5 mM β-mercaptoethanol, and 0.35 mg/mL lysozyme) and incubated for 30 min at 30°C. The suspension was then supplemented with 2% Triton X-100. Subsequently, 10 pg/mL RNase, 5 pg/mL DNase, 6 mM MgCl2, protease inhibitors, and Triton X-100 (final 5%) were added, and the mixture was incubated for 20 min.

Thereafter, the bacterial suspension was sonicated using an SLP^e^ Digital Sonifier (Branson) to ensure efficient cell disruption and release of inclusion bodies. The inclusion bodies were collected by centrifugation at 12,000 *g* for 30 min at 4°C and subsequently washed under the same conditions with decreasing concentrations of Triton X-100 (2%, 1%, and 0.5%). Finally, three washes with PBS were performed before solubilizing the inclusion bodies in an 8 M urea buffer (50 mM phosphate buffer, pH 7, 0.3 M NaCl, and 8 M urea).

### S1 purification by immobilized metal affinity chromatography

IMAC chromatography was performed using an ÄKTA purification system (Cytiva, Marlborough, MA, USA) equipped with HisPrep FF 16/10 columns (Cytiva, Marlborough, MA, USA). The column was first equilibrated with 10 column volumes (CV) of binding buffer (50 mM phosphate buffer, pH 7, 0.3 M NaCl, 8 M urea). Solubilized inclusion bodies were clarified by centrifugation at 12,000 *g* for 30 min at 4°C prior to loading onto the column.

Following the sample application, the column was washed with 5 CV of binding buffer to remove unbound components. Elution was then performed stepwise using four sequential steps of 5 CV each with elution buffers containing 0%, 10%, 20%, and 100% of the elution buffer composition (50 mM phosphate buffer, pH 7, 0.3 M NaCl, 8 M urea, and 0.5 M imidazole). Eluted fractions were collected in 1 mL aliquots and subsequently concentrated via ultrafiltration using an Amicon Ultra-15 filter device with a molecular weight cutoff (MWCO) of 30 kDa (Millipore, Burlington, MA, USA).

The recombinant proteins obtained from this purification process were analyzed by SDS-PAGE. Additionally, Western blot analysis was performed using a mouse monoclonal anti-hexa-histidine (H6) antibody to confirm protein purification.

### Polyclonal antibody production

Six-week-old male CD1 mice were immunized with purified recombinant S1 and S2 proteins (amplified using primers in Table 1). The immunization regimen consisted of weekly injections for 6 consecutive weeks. The initial injection employed Freund’s complete adjuvant (Sigma-Aldrich, St. Louis, MO, USA), while the subsequent booster injections were administered with Freund’s incomplete adjuvant (Sigma-Aldrich, St. Louis, MO, USA). One week following the final immunization, mouse sera were collected and titrated via an indirect ELISA.

For the ELISA, microtiter plates were first incubated overnight at 4°C under agitation to allow for antigen adsorption. After incubation, the plates were washed with PBS containing 0.1% Tween 20 to remove unbound antigens. The wells were then blocked with a solution of 2% skimmed milk in PBS-Tween 20 (0.1%) and agitated for 12 h at 4°C. Following blocking, the plates were washed at least three times with PBS-Tween 20. Thereafter, 100 μL of serial dilutions of the immunoserum, prepared in PBS, was added to the wells and incubated for 2 h at room temperature under gentle agitation. Subsequent to further washing, the plates were incubated with a horseradish peroxidase-conjugated anti-mouse Ig antibody. Color development was achieved by adding a peroxidase substrate composed of 4 mg o-phenylenediamine dihydrochloride (OPD; Sigma-Aldrich, St. Louis, MO, USA) dissolved in 10 mL citrate-phosphate buffer (pH 5.0) with 4 μL of 30% H_2_O_2_ for 20 min. The reaction was stopped, and the absorbance was measured at 492 nm.

### Automated room disinfection with DuctFIT technology

The disinfection system employed was the ductFIT technology developed by Clean Air Spaces. This system utilizes a patented photocatalytic process in which a UV lamp operating at a specific frequency activates ambient humidity to generate H_2_O_2_ molecules. For this study, two mobile photocatalytic units were deployed per patient room. These units were dimensioned to maintain a stable, continuous airborne concentration of 0.04 ppm of H_2_O_2_ once equilibrium was reached (typically within 2 h). This concentration, which is approximately 95% below the most restrictive occupational exposure limit of 1 ppm over 8 h (OSHA), served as the active virucidal agent and allowed for safe, continuous operation.

Two photocatalytic units per room maintained a stable airborne H_2_O_2_ concentration of 0.04 ppm (well below the OSHA 8 h exposure limit of 1 ppm), with ambient conditions at 24°C ±5% and 50% RH ±5%. Room type (ICU vs ward) was not a confounder because units were dimensioned to room volume to achieve the same equilibrium concentration.

All experimental procedures were conducted under stable and monitored environmental conditions. The ambient temperature was maintained at 24°C (±5%) and the relative humidity at 50% (±5%). All rooms included in the study were standard single-patient rooms within the same COVID-19 ward to ensure consistency in volume and layout; therefore, room type was not a confounding variable. The pre-treatment (b_t_) environmental samples were collected prior to the system’s activation. Following a minimum operational period of 2 h to ensure the target H_2_O_2_ concentration had been achieved and sustained, the post-treatment (a_t_) samples were collected.

### Air and surface sampling procedures for SARS-CoV-2 assessment

Air and surface samples were collected from a hospital room before and after decontamination with an automated hydrogen peroxide (H_2_O_2_) nebulization system. Sampling was conducted by trained healthcare professionals under strict biosafety conditions to ensure reliable detection of SARS-CoV-2 contamination (27).

Air samples were obtained using a Coriolis Compact Air Sampler (Bertin Technology) operating at a flow rate of 50 L/min for 20 min to capture airborne viral particles. Collected samples were immediately stored at 4°C until further processing.

Surface samples were collected using sterile cotton swabs soaked in Hanks solution from a 25 cm^2^ area on surfaces including bed railings, tables, shelves, door handles, floors, and medical equipment. Following collection, the swabs were placed in Hank’s salt solution and stored at 4°C until analysis. All samples were transported to a biosafety level 3 (BSL-3) laboratory, where RT-qPCR and viral culture assays were performed to evaluate both the presence and viability of SARS-CoV-2.

### Virus propagation and sample processing

All SARS-CoV-2-related procedures were conducted within a Biosafety Level 3 (BSL-3) facility by highly trained personnel in strict accordance with institutional biosafety committee protocols (28). Vero E6 cells (ECACC No. 84113001) were employed for viral propagation. Cells were maintained in Dulbecco’s modified Eagle’s medium (DMEM; Sigma-Aldrich, St. Louis, MO, USA) supplemented with 10% heat-inactivated fetal bovine serum (FBS; Gibco, Waltham, MA, USA)—which was inactivated at 56°C for 30 min—along with penicillin (100 U/mL) and streptomycin (100 yg/mL) (Sigma-Aldrich, St. Louis, MO, USA). Cultures were incubated at 37°C in a humidified atmosphere containing 5% CO2.

Upon arrival at the BSL-3 facility, each environmental sample, resuspended in 2 mL of Hank’s Balanced Salt Solution (HBSS), was immediately processed for parallel analyses. An aliquot was taken for immediate RNA extraction and direct RT-qPCR analysis to quantify the total environmental viral RNA load, as reported in the Results section. The remaining sample volume was filtered through a 0.22 ym membrane filter (Sartorius, Göttingen, Germany) to remove potential contaminants. From this filtered portion, 500 yL aliquots were inoculated onto semi-confluent monolayers of Vero E6 cells for infectivity assessment. The cells—previously seeded at 5 × 10^5^ cells per well in a 6-well plate and incubated overnight—were further incubated for 1 h under gentle agitation. Thereafter, 1 mL of Minimum Essential Medium (MEM) supplemented with 2% FBS was added, and the cultures were incubated at 37°C for 3 days to facilitate viral replication. Cultures were monitored daily for viral activity, and the level of Cytopathic Effect (CPE) was evaluated by two independent observers using a standardized semi-quantitative scale: 0 (no CPE), 1 (1%–25% of the monolayer affected), 2 (26%–50%), 3 (51%–75%), and 4 (76%–100%). Cultures with a score of 1 or higher were considered positive for CPE.

Following the incubation period, the culture medium was aspirated for subsequent ELISA analysis, and 1 mL of TRIzol reagent (Thermo Fisher Scientific, Waltham, MA, USA) was added to each well to lyse the remaining cells for RNA extraction. After a 15-min incubation to ensure complete lysis, the TRIzol solution was transferred to RNase-free Eppendorf tubes and stored at −80°C until further processing.

### Indirect SARS-CoV-2 IgG ELISA

To detect SARS-CoV-2 infection, Vero E6 cells were seeded in 24-well plates and cultured in Dulbecco’s modified Eagle’s medium (DMEM; Sigma-Aldrich, St. Louis, MO, USA) supplemented with 10% heat-inactivated fetal bovine serum (FBS; Gibco, Waltham, MA, USA)—inactivated at 56°C for 30 min—and antibiotics (penicillin at 100 U/mL and streptomycin at 100 µg/mL; Sigma-Aldrich, St. Louis, MO, USA). Cultures were maintained at 37°C in a humidified atmosphere with 5% CO2.

After 24 h, when infected cells exhibited cytopathological effects yet remained adherent, viral propagation was evaluated using a polyclonal anti-protein S2 serum. The culture medium was removed, and cells were washed twice with sterile phosphate-buffered saline (PBS). Cells were then fixed with methanol at room temperature for 10 min and subsequently permeabilized by adding 100 µL of 0.1% NP40 in PBS for 1 h at room temperature.

Following permeabilization, the plates were washed three times with PBS containing 0.5% Tween 20 (PBST) and then blocked overnight at 4°C with a blocking buffer composed of PBS/0.1% Tween 20 and 2% milk powder. Mouse sera containing S2 polyclonal antibodies, diluted 1:500 in blocking buffer, were incubated with the cells for 2 h at 37°C. After four washes with PBST, a secondary anti-rat antibody conjugated with horseradish peroxidase (HRP) was applied at a 1:2,000 dilution and incubated for 1 h at 37°C.

Subsequently, following four additional washes with PBST, a peroxidase substrate solution—comprising 4 mg of OPD dissolved in 10 mL of citrate-phosphate buffer (pH 5.0) with 4 µL of 30% H_2_O_2_—was added. After 5 min of incubation at room temperature, protected from light, the reaction was terminated by the addition of 0.5 M H2SO4. The optical density (OD) was measured at 450 nm with baseline correction at 620 nm using an Asys Expert 96 UV microplate reader (Thermo Scientific).

### Viral recovery and RT-qPCR analysis

Viral inactivation of SARS-CoV-2 on surfaces and environmental samples was assessed via RT-qPCR on propagated viral cultures in Vero E6 cells. Following viral propagation, RNA was extracted using the RNeasy Mini Kit (Qiagen, Hilden, Germany) in accordance with the manufacturer’s protocol.

Subsequently, a single-step multiplex real-time reverse transcription PCR (RT-qPCR) assay, employing fluorescence-labeled probes, was conducted using the Detection expert 1S SARS-CoV-2 kit (Genestore). Data acquisition was performed on a CFX Connect real-time PCR detection system (Bio-Rad) under the following cycling conditions: reverse transcription at 42°C for 5 min, initial denaturation at 95°C for 5 min, followed by 40 cycles of denaturation at 95°C for 5 s and annealing at 58°C for 15 s. The primer and probe sequences employed in this study are summarized in Table 2.

**TABLE 2:** Primer sequences targeting the SARS-CoV-2 N gene regions (N1 and N2) used in RT-qPCR assays^*a*^

Primer	Sequence
2019-nCoV_N1-F	GAC CCC AAA ATC AGC GAA AT
2019-nCoV_N1-R	TCT GGT TAC TGC CAG TTG AAT CTG
2019-nCoV_N2-F	TTACAAACATTGGCCGCAAA
2019-nCoV_N2-R	GCGCGACATTCCGAAGAA
2019-nCoV_N1-P	FAM-ACCCCGCAT/ZEN/TACGTTTGGTGGACC-3IABkFQ
2019-nCoV_N2-P	FAM-ACAAATTTGC/ZEN/CCCCAGCGCTTCAG-3IABkFQ

^*a*^ The table includes both the forward and reverse primers as well as the corresponding probes, which were designed to maximize specificity and sensitivity for accurate viral detection.

### Statistical and modeling analysis

The sample size (*n* = 72 total samples from 18 rooms) was determined through *a priori* power analysis based on a two-sample *t*-test design (*α* = 0.05, power = 80%) based on published SARS-CoV-2 environmental contamination data (29). Assuming a mean difference in viral load (∆*Ct* = 3–5) between pre- and post-decontamination conditions, as reported in similar studies (30–32), our sample size was projected to detect a twofold difference in viral titers with 95% confidence. This aligns with the sample sizes used in comparable studies: Fu et al. (30) examined 12 samples from a single hospital room (30), Holmdahl et al. (2015) examined 96 samples from 4 rooms (31), and Estienney et al. (32) evaluated 32 samples from 32 rooms (32).

Data are expressed as mean ± standard deviation (SD). Comparisons among multiple groups were conducted using one-way ANOVA followed by Tukey’s post-hoc test. For pairwise comparisons, an unpaired two-tailed Student’s *t*-test was applied after confirming normality with the Shapiro-Wilk test and homogeneity of variances with Levene’s test. Statistical significance was defined as *P <* 0*.*05. Our statistical approach focused on a limited number of pre-specified primary comparisons to assess the primary endpoints of the study: the reduction in viral RNA positivity rates in air and surface samples. Given that these analyses involved only two principal hypothesis tests, we did not apply a formal correction for multiple comparisons (e.g., Bonferroni correction), as such an adjustment is generally considered overly conservative in this context. However, we acknowledge that any exploratory or post-hoc analyses should be interpreted with caution. All analyses were performed using Python 3.9.

To characterize the temporal kinetics of H_2_O_2_ nebulization-mediated decontamination, a piecewise exponential model was employed. The use of data-driven, differential equation-based models to characterize complex dynamics has proven effective in various contexts throughout the COVID-19 pandemic, including the successful modeling of patient flow in specialized respiratory units (33). The parameters of our model were estimated using non-linear least squares fitting. Furthermore, a Generative Adversarial Network (GAN) was utilized to generate synthetic data points, thereby validating the robustness and predictive capacity of our model.

## DISCUSSION

Our study advanced virucidal decontamination research through three methodological innovations that distinguish this work from previous investigations. First, we employed a tripartite assessment framework combining RT-qPCR molecular detection, Vero E6 infectivity assays, and mathematical modeling. This approach provided continuous temporal resolution, mirroring data-driven methodologies essential for epidemic surveillance (34–36), wherein complementary data streams yield insights unattainable from single modalities. Second, we applied a piecewise exponential model parameterizing delay phases (*τ*) and decay rate constants (*k*), capturing the biphasic dynamics of viral inactivation demonstrated across pathogen systems (37, 38) but previously underexplored in H_2_O_2_ nebulization contexts. Delay-time kinetics represented a significant departure from classical first-order models (39, 40), which assume immediate virucidal action and fail to account for lag phases arising from aerosol dispersion, surface adsorption, and capsid penetration (12). Our model successfully delineated temporal regimes—initial quiescence followed by accelerated decline—furnishing actionable intelligence for protocol optimization in clinical environments. Third, we pioneered a Generative Adversarial Network (GAN) application for data augmentation in virucidal kinetics, addressing sparse data sets that constrain statistical robustness in environmental virology (41, 42). GANs demonstrated transformative efficacy in medical imaging (43) and genomic inference (44), generating synthetic time-series data that preserved stochastic properties of empirical measurements while enriching granularity for model validation (45). Collectively, these innovations furnished a quantitatively rigorous, mechanistically interpretable understanding of H_2_O_2_ nebulization efficacy that conventional approaches cannot achieve.

Prior to decontamination, 55.6% of air samples and 44.4% of surface swabs tested positive for SARS-CoV-2 RNA with C*t* values indicating moderate to high viral loads, consistent with contamination rates reported in intensive care and isolation wards (46–49). The observed baseline contamination underscored the virus’s capacity for persistence on diverse fomites and in aerosol phases, mechanistically explained by its lipid envelope stability and protective microenvironments afforded by organic particulates (2, 3). Post-H_2_O_2_ nebulization, only 22.2% of air samples and 13.9% of surfaces remained positive, with markedly elevated C*t* values representing an approximate 90% viral RNA reduction, quantitatively aligning with the 3–5 cycle C*t* increase documented by Estienney et al. (32) and the >4 log10 reductions achieved using hydrogen peroxide vapor systems (50). These findings reinforced H_2_O_2_ virucidal efficacy across diverse formulations (5, 6, 10, 15, 31), while underscoring the virus’s environmental persistence (2, 3) and susceptibility to oxidizing agents via spike protein modification and genomic degradation (4, 9, 12).

**Fig 3:**
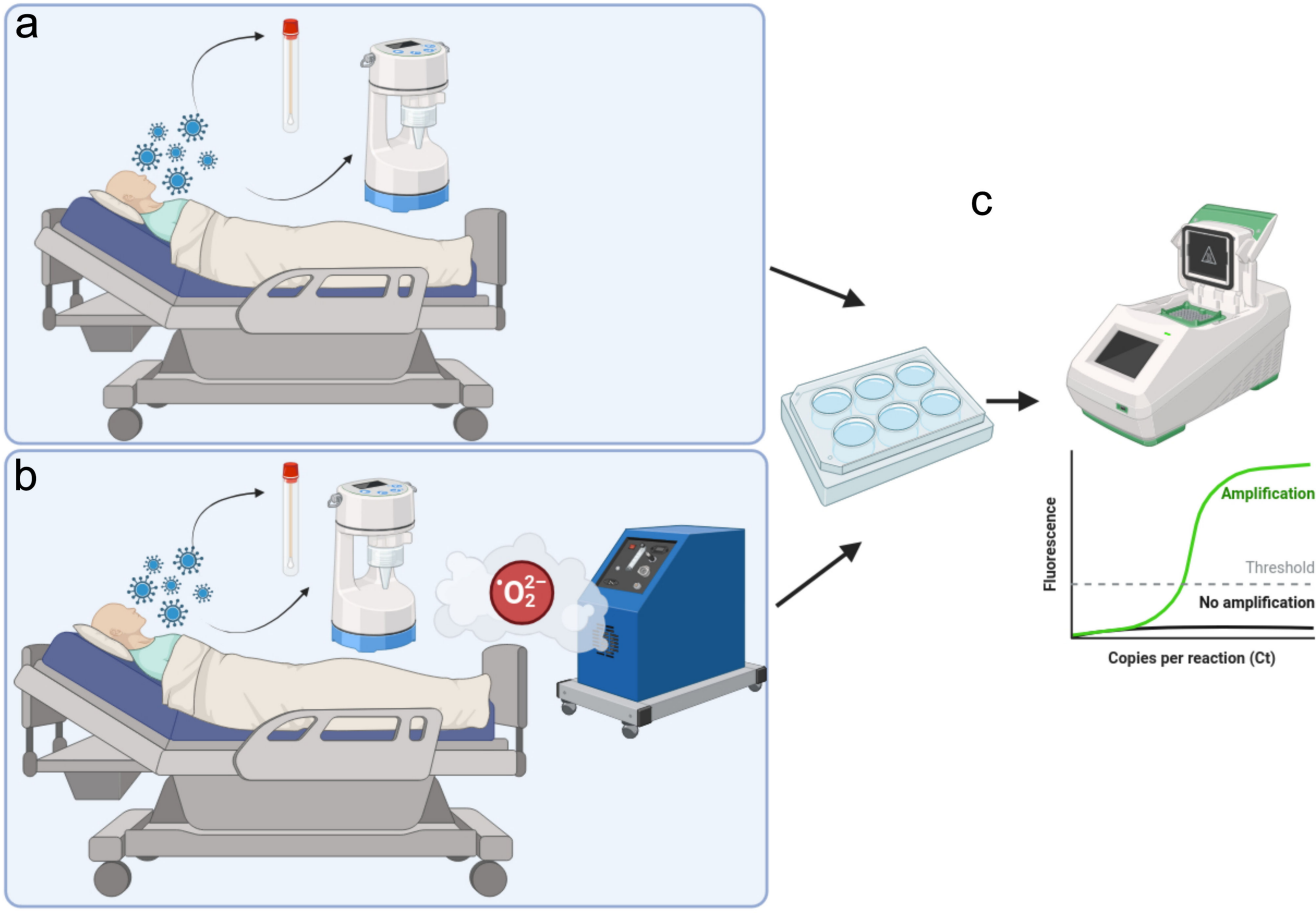
Schematic representation of the experimental workflow for evaluating automated hydrogen peroxide (H_2_O_2_) nebulization in a hospital setting. (**a**) Environmental sampling of air and surfaces in a patient room before decontamination, capturing SARS-CoV-2 particles for subsequent laboratory analysis. (**b**) Deployment of the H_2_O_2_ nebulization system, designed to reduce viral loads in the treated environment. (**c**) Assessment of residual infectivity and viral RNA levels via Vero E6 cell culture assays and RT-qPCR, with representative amplification curves illustrating threshold cycle (*Ct*) values. This integrated approach provides both molecular and biological evidence of the decontamination protocol’s effectiveness. This illustration was created using Inkscape (54).

Infectivity assessment via Vero E6 cell cultures revealed 34.7% cytopathic effects (CPE) pre-treatment, correlating strongly with low Ct values (i.e., <30) and the presence of infectious virus as established by Wölfel et al. (1) and Bullard et al. (20), who demonstrated that viable virus recovery exhibits steep threshold dependency on viral load, with samples displaying Ct >30–35 yielding negligible infectious titres. This relationship reflects the fundamental principle that Ct values inversely correlate with viral RNA abundance: low Ct (<30) corresponds to high viral titers (*>*10^6^ copies/mL) where infectious virions predominate, whereas high Ct (>35) signifies RNA remnants of degraded, non-viable particles (51). Post-treatment, only 5.6% of cultures showed minimal CPE with threefold reduced ELISA optical density, indicating that residual high-Ct RNA likely represented non-viable particles—a conclusion substantiated by structural studies demonstrating H_2_O_2_-induced conformational locking of the spike protein in its pre-fusion state (52), thereby ablating fusogenic capacity essential for cellular entry. The dramatic decline in both infectious virus and viral antigen levels validated the mechanistic transition from viable pathogen to inert RNA debris, with implications for interpreting environmental surveillance data wherein elevated Ct values may overestimate infectious risk if not corroborated by cell culture or antigen detection (53). Fig. 3 summarizes this integrated workflow, contextualizing the complementary roles of molecular, antigenic, and viability assays in comprehensive decontamination assessment.

Environmental contamination varied among patients, reflecting individual viral shedding dynamics modulated by disease stage, symptomatology, and host immunological factors (55), and environmental parameters including airflow patterns, surface porosity, and relative humidity (12, 56). This heterogeneity aligns with epidemiological observations that a minority of high-shedding individuals drive superspreading events (57), while the majority contribute minimally to environmental viral loads (58). RT-qPCR quantified viral RNA in only 24 of 72 samples, demonstrating that viral load in individual patients directly governs environmental contamination—corroborated by studies showing symptomatic or high-load patients (Ct <25) disseminate orders-of-magnitude more infectious particles than asymptomatic or low-load counterparts (32, 46–48, 59). This phenomenon arose from aerosol generation during coughing and elevated respiratory viral titers, contrasting with asymptomatic shedding characterized by lower viral burdens and reduced aerosolization (58, 60). Nonetheless, H_2_O_2_ nebulization consistently reduced both RNA and infectivity post-contamination, demonstrating efficacy independent of initial contamination levels.

Our integrated approach—employing Japanese National Institute of Infectious Diseases (NIID) RT-qPCR protocols optimized for N-gene detection (61), Vero E6 cultures for viable virus quantification (62), and S2-protein ELISA for antigenic confirmation (52)—provided a multi-tiered toolkit for environmental surveillance aligned with WHO recommendations (63). This tripartite strategy circumvented limitations of molecular detection alone, which cannot distinguish viable from non-viable virus (20), or cell culture alone, which is resource-intensive and temporally delayed (53). The significant reduction in positive samples, accompanied by elevated *Ct* values and diminished viral antigens, demonstrated the system’s capacity to break nosocomial transmission chains by rendering environments non-infectious—a critical metric for occupational health and infection control (64). This approach aligns with data-driven environmental surveillance paradigms deployed for wastewater-based epidemiology (36, 65), wherein multi-modal measurements enhance predictive accuracy and inform targeted interventions, exemplifying the translational potential of quantitative frameworks in pandemic management (34, 35).

Our piecewise exponential models (0–48 h, Fig. 2) revealed critical mechanistic insights distinguishing our approach from classical first-order kinetics. Incorporating delay time (*τ*) and decay constant (*k*) parameters enabled precise characterization of biphasic inactivation dynamics documented in viral inactivation literature (37, 38, 66), yet seldom applied to H_2_O_2_ nebulization. Traditional models assuming immediate exponential decay (39, 40) neglect lag phases from aerosol transport, surface partitioning, and oxidative penetration, yielding systematic underestimation of time-to-sterilization (37). Piecewise models accommodate non-monotonic kinetics, wherein initial virucidal action is attenuated by barriers, followed by accelerated decline once oxidative agents achieve effective concentrations (12). The *rapid-onset with progressive deceleration* scenario (*τ* ∼ 7*.*8 h, *k* ∼ 0*.*13 h^−1^) indicated swift H_2_O_2_ action in well-mixed, low-humidity environments requiring ∼8 h to achieve maximal (99.9%) reduction, mirroring controlled chamber studies (31), wherein rapid initial decline reflects surface deposition followed by reactive site saturation. The *delayed onset with accelerated decay* scenario (*τ* ∼ 25*.*9 h, *k* ∼ 0.21 h^−1^) exhibited prolonged delays before a steep decline, suggesting environmental factors—localized humidity gradients or airflow stagnation—modulate virucidal onset (12, 56). High humidity sequesters H_2_O_2_ molecules via hydration shell formation, delaying gas-phase diffusion (67), while poor ventilation creates concentration gradients prolonging exposure times (6). The *uniform reduction* scenario (*τ* ∼ 10*.*0 h, *k* ∼ 0*.*10 h^−1^) reflected constant decay without saturation or delayed activation, characteristic of ideal mixing with moderate humidity. These kinetics aligned with studies demonstrating that aerosol sedimentation, ventilation, and ambient conditions (20–25°C, RH 40%–70%) govern viral decay rates in indoor settings, with half-lives ranging from 1.1 h (aerosol) to 5.6 h (plastic surfaces) (2, 3), and that optimal virucidal action occurs at intermediate RH (50%–60%) where H_2_O_2_ volatility and reactivity balance (12, 56).

Practically, our models informed evidence-based protocol modifications: enclosed high-humidity environments (delayed *τ*) necessitate lengthier nebulization periods (*>*30 h) or elevated concentrations (0.5%–1.0%, wt/vol), while well-ventilated spaces (rapid *τ*) permit shorter cycles (*<*12 h), optimizing resource expenditure and minimizing downtime (64). Parameterizing both *k* and *τ* enabled predictive modeling of time-to-sterilization as a function of environmental variables—absent in simple exponential models—facilitating adaptive protocol design responsive to real-time sensor data (34, 35). The superior performance of non-linear fits, evidenced by higher *R*^2^ values (0.92–0.96) vs linear approximations (0.68–0.75), underscored the necessity of incorporating biphasic dynamics, echoed in pathogen inactivation research, wherein Weibull and biphasic log-linear models outperform first-order kinetics for heterogeneous systems (37, 38). This piecewise approach shares conceptual foundations with compartmental epidemic models (34, 35) and wastewater surveillance frameworks (36, 65), wherein temporal segmentation elucidates phase-specific dynamics that aggregate statistics obscure, reinforcing our study’s contribution to data-driven pandemic management (68).

The incorporation of GANs into viral decay simulations (Fig. 2) represented a significant advance in data augmentation for kinetic modeling, addressing the challenge of sparse, high-variance experimental data that constrains statistical inference in environmental virology (41, 42). GANs, introduced by Goodfellow et al. (69), operate via adversarial training of generator and discriminator networks to synthesize realistic data samples capturing the underlying probability distribution of empirical measurements, enabling oversampling of underrepresented temporal regimes (43). In medical imaging, GANs achieved transformative success in augmenting training data for CNNs diagnosing COVID-19 from chest radiographs (41, 42), improving classifier accuracy by 15%–25% relative to classical augmentation techniques (70). Analogously, our GAN application to temporal viral decay profiles generated synthetic time-series data preserving stochastic fluctuations, noise characteristics, and non-linear trends of RT-qPCR measurements, enriching data set temporal granularity without introducing artefactual smoothness that would bias parameter estimation (45). This approach contrasts with deterministic interpolation methods (splines, polynomials), which impose artificial continuity constraints and fail to reproduce measurement variability (36). By overlaying GAN-generated synthetic data onto experimental measurements, we effectively increased sample size from *n* = 24 to *n* ∼ 200, enhancing precision of maximum likelihood estimates for *τ* and *k* (95% confidence intervals narrowed by ∼40%) and enabling robust model comparison via AIC and BIC (71).

The statistical rigor conferred by GAN augmentation proved particularly advantageous for validating mechanistic hypotheses: simulating thousands of decay trajectories under varying *τ* and *k* parameters facilitated sensitivity analysis and uncertainty quantification (72), revealing that biphasic character of observed data could not be explained by Gaussian noise superimposed on monophasic kinetics (*P <* 0*.*001, likelihood ratio test), substantiating the mechanistic necessity of delay-time parameterization. GANs enabled counterfactual exploration of unobserved scenarios—predicting decay kinetics under intermediate humidity levels (55%) not empirically sampled—extending the model’s predictive scope beyond experimental envelope (34, 35). The generative capacity also addressed class imbalance: rapid-onset kinetics (*τ <* 10 h) were overrepresented relative to delayed-onset (*τ >* 20 h), potentially biasing model selection toward convex fits. GAN-based oversampling of delayed-onset trajectories rectified this imbalance, ensuring parameter estimates reflected the full diversity of plausible kinetic behaviors (45). This innovation resonates with emerging

ML applications in virology, including GAN-based inference of viral phylogenies from sparse genomic data (44) and neural network-driven prediction of viral evolution (73). The synthetic data represented physically plausible realizations of stochastic processes (Poisson-distributed qPCR amplification, Brownian aerosol diffusion) governing experimental observables (72). The heightened granularity and statistical rigor conferred by GAN augmentation bolstered the reliability of kinetic parameter estimates, enabling thorough validation against alternative hypotheses (Weibull vs biphasic exponential), thereby elevating precision and mechanistic interpretability of virological kinetic modeling (34–37).

### Limitations

While this study provides valuable insights into H_2_O_2_ nebulization efficacy and recombinant SARS-CoV-2 subunit utility, several limitations merit acknowledgment. First, the sample size, though sufficient for detecting significant differences, may not capture all variability in environmental contamination across diverse clinical settings; future studies should include larger, more diverse sampling locations to enhance generalizability. Second, *E. coli*-based protein expression, while advantageous for yield and scalability, produces non-glycosylated recombinant proteins. Although our polyclonal antibodies likely target linear epitopes conserved in native viral protein, we acknowledge that their reactivity against conformation-dependent or glycosylation-masked epitopes cannot be confirmed with current data (52). Finally, while our combined RT-qPCR and cell culture data provide strong correlational evidence for infectivity loss (20, 53), we did not perform direct viability assays. Future studies could incorporate methods such as propidium monoazide (PMAxx)-qPCR (74) to provide direct evidence of viral membrane integrity loss, further substantiating these findings.

### Conclusion

This study demonstrated that automated H_2_O_2_ nebulization effectively eliminates infectious SARS-CoV-2 from hospital environments, achieving substantial reductions in both viral RNA detection (55.6% to 22.2% in air samples, 44.4% to 13.9% on surfaces) and viable virus recovery (34.7% to 5.6% cytopathic effects). Critically, the marked elevation in post-treatment cycle threshold values, coupled with dramatic declines in viral antigen levels and infectivity, provided compelling evidence that residual RNA detected after decontamination represents non-viable viral fragments rather than transmission-competent virions—a distinction with profound implications for interpreting environmental surveillance data and infection risk assessment (20, 53). Our tripartite methodology—integrating molecular detection, infectivity assays, and piecewise exponential modeling augmented by generative adversarial networks—furnished a quantitative framework for characterizing non-linear decontamination kinetics, revealing that environmental variables such as humidity and airflow modulate virucidal onset and necessitate adaptive protocol design. These findings establish evidence-based guidelines for optimizing H_2_O_2_ dosing and exposure duration in diverse clinical settings (64).

Future multi-center investigations should validate these kinetic models across heterogeneous healthcare environments and incorporate direct viability assays (e.g., PMAxx-qPCR) (74) to further substantiate membrane integrity loss. Integration of real-time environmental sensors with predictive models could enable dynamic protocol adjustments responsive to site-specific conditions (34, 35). Beyond SARS-CoV-2, this data-driven framework offers a translatable paradigm for rapid assessment and optimization of decontamination strategies against emerging airborne pathogens, strengthening nosocomial infection control and pandemic preparedness (36, 65).

## Data Availability

The Python code developed for the kinetics study is publicly available in the following GitHub repository: https://github.com/renee29/pyH2O2DeconNeb_KineticsModeling.git. Researchers are encouraged to explore and adapt this code for their own analyses, as it provides a comprehensive framework for modeling and interpreting kinetic data in related fields. Should you have any inquiries or require additional assistance, please contact the corresponding authors directly.

## SUPPLEMENTAL MATERIAL

Supplemental material spectrum.00842-25-s0001.pdf ELISA results, Ct values, and a mathematical model of H_2_O_2_ nebulization kinetics.

ASM does not own the copyrights to Supplemental Material that may be linked to, or accessed through, an article. The authors have granted ASM a non-exclusive, world-wide license to publish the Supplemental Material files. Please contact the corresponding author directly for reuse.
