# Supplementary material for: Exploring novel kinetics of automated H2O2 nebulization: a breakthrough in SARS-CoV-2 elimination
Source: Microbiol Spectr. 2026 Apr 3;14(5):e00842-25. doi: 10.1128/spectrum.00842-25 (PMC13141947; doi:10.1128/spectrum.00842-25)
Supplement: Supplemental material — ELISA results, Ct values, and a mathematical model of H2O2 nebulization kinetics. [file spectrum.00842-25-s0001.pdf]

Supporting Information: Exploring Novel Kinetics of Automated H_2O_2 Nebulisation: A Breakthrough in SARS-CoV-2 Elimination.

Jennifer Solano-Parada[‡,1], José Antonio Sánchez-Martínez[‡,2,3], Beatriz Carolina Gómez-Hernández[2,3], Margarita Barriga[1], Pilar García-Velasco[2], Alberto Fernández[1], Alberto Cornet-Gómez[1], Antonio Osuna Carrillo de Albornoz[‡,1], Rene Fabregas[‡,*,4], and Concepción Morales-García[‡,2,3]

[1]Institute of Biotechnology, Department of Parasitology, CTS183 Group, University of Granada, Granada, Spain.
[2]Department of Pneumology, University Hospital Virgen de Las Nieves, Granada, Spain.
[3]Biosanitary Research Institute of Granada-Ibs, Granada, Spain.
[4]Department of Applied Mathematics and Modeling Nature (MNat) Research Unit, Faculty of Sciences, University of Granada, Granada, Spain.

[4]**Corresponding Author**: *Rene Fabregas, rfabregas@ugr.es.

Tuesday 18th November, 2025

1 Validation of Recombinant SARS-CoV-2 S2 Protein Expression

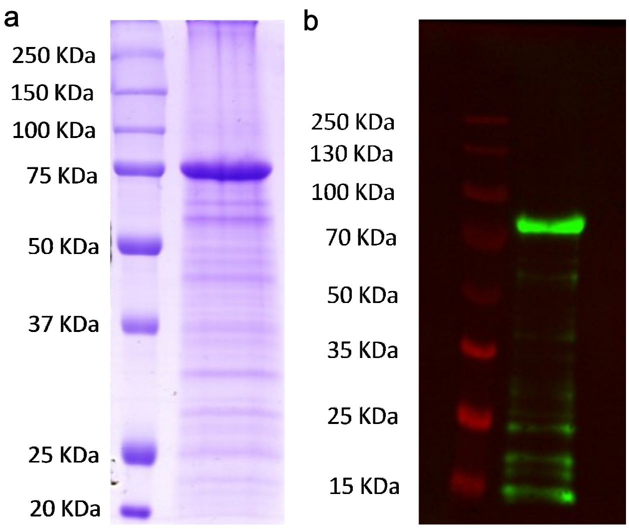

Figure S1 | Verification of Recombinant S2-His6 Protein Expression. (a) Coomassie-stained SDS-PAGE of the purified protein. The right lane shows a distinct band at the expected molecular weight of 75 kDa, compared to a protein ladder (left lane). **(b)** Western blot confirmation. The protein was detected using an anti-His6 monoclonal antibody, revealing a specific fluorescent band (green) at 75 kDa, confirming its identity.

The successful expression and purification of the recombinant SARS-CoV-2 S2 subunit, used for the generation of polyclonal antibodies for the ELISA assays, was confirmed prior to immunization. As shown in Figure S1, both SDS-PAGE and Western blot analyses confirmed the presence of a protein of the correct molecular weight (75 kDa) with the expected His6 tag.

2 ELISA OD Measurements versus Control.

Table S1 presents representative ELISA results, reporting optical density (OD) values relative to a control and their corresponding standard deviations (SD). In our study, air samples from patients

,‡ These authors contributed equally.

were assayed both before and after H_2O_2 nebulisation. Notably, Patient 2's pre-treatment air sample exhibited a high OD of 0.7443 ± 0.3734, which decreased to 0.3196 ± 0.0428 following treatment—consistent with RT-qPCR data indicating a reduction in viral load. Patient 1, while showing a more modest decline (from 0.1639 ± 0.3750 to 0.1420 ± 0.2698), similarly demonstrated reduced antigen presence post-treatment. These findings substantiate the efficacy of H_2O_2 nebulisation and align with previous reports [1, 2].

Sample	OD vs. Control ± SD
Patient 1 – Air (No H_2O_2)	0.1639 ± 0.3750
Patient 1 – Air (With H_2O_2)	0.1420 ± 0.2698
Patient 2 – Air (No H_2O_2)	0.7443 ± 0.3734
Patient 2 – Air (With H_2O_2)	0.3196 ± 0.0428

Table S1 | Representative ELISA OD measurements versus control and corresponding standard deviations (SD) from S2-based assays, conducted before and after H_2O_2 nebulisation.

3 Duplicate ELISA Measurements.

Table S2 presents a subset of duplicate ELISA measurements of optical density (OD) values, recorded as "Duplicate 1" and "Duplicate 2", for samples collected from patients both before (bt) and after (at) H_2O_2 nebulisation. These replicate measurements are crucial for assessing the intra-assay precision and reproducibility of the ELISA method used to quantify SARS-CoV-2 antigen levels. For example, Patient 1's untreated air sample yielded OD values of 1.4277 and 0.8973, while after treatment, the values decreased to 1.3314 and 0.9499. Similarly, Patient 2's air sample prior to treatment exhibited an OD of 2.0070 ± 1.4789, which decreased to 1.3485 ± 1.2879 following treatment—an observation that aligns with the documented reduction in viral infectivity. An additional "Extra case" is included as a reference control. Such reproducibility in the OD readings supports the reliability of our assay, consistent with the methodologies reported in the literature [3, 4]. A detailed statistical summary is provided in Table S2.

Sample	Duplicate 1	Duplicate 2
Patient 1 – Air (No H_2O_2)	1.4276796	0.8973299
Patient 1 – Air (With H_2O_2)	1.3314332	0.9498714
Patient 2 – Air (No H_2O_2)	2.0070044	1.4788834
Patient 2 – Air (With H_2O_2)	1.3485021	1.2879408
(Extra case)	1.017415211	0.979823574

Table S2 | Duplicate ELISA Measurements for Air Samples. This table presents replicate optical density (OD) readings for air samples collected from patients before and after H_2O_2 nebulisation.

4 Cycle Threshold (C_t) values for Patients P1–P6.

Table S3 presents a subset of Cycle Threshold (C_t) measurements (reported as mean ± standard deviation (SD)) for six patients (P1–P6) under various sampling conditions. In the table, "A" denotes air samples and "S" denotes surface swabs, with "bt" indicating samples collected before treatment and "at" after H_2O_2 nebulisation. Notably, several conditions exhibit undetectable viral RNA (recorded as 0), particularly post-treatment, which is consistent with a marked reduction in viral load.

For example, patient P1's data show a C_t value of 25.00 ± 1.41 in the air sample before treatment, which increases to 29.42 ± 0.68 after treatment, while surface samples remain undetectable. Similarly, patient P3 demonstrates a C_t of 29.82 ± 1.45 for surface swabs before treatment that increases to 34.15 ± 4.53 after treatment. These findings underscore the efficacy of H_2O_2 nebulisation in reducing detectable viral RNA, in agreement with our broader observations.

Patient	Condition	$C_t \pm$ SD
P1	Abt	25.00 ± 1.41
P1	Aat	29.42 ± 0.68
P1	Sbt	0.00 ± 0.00
P1	Sat	0.00 ± 0.00
P2	Abt	23.48 ± 0.68
P2	Aat	0.00 ± 0.00
P2	Sbt	31.87 ± 1.41
P2	Sat	0.00 ± 0.00
P3	Abt	25.02 ± 0.08
P3	Aat	26.32 ± 2.10
P3	Sbt	29.82 ± 1.45
P3	Sat	34.15 ± 4.53

Patient	Condition	$C_t \pm$ SD
P4	Abt	26.31 ± 0.92
P4	Aat	29.93 ± 0.09
P4	Sbt	33.46 ± 3.53
P4	Sat	34.90 ± 2.79
P5	Abt	36.39 ± 3.40
P5	Aat	0.00 ± 0.00
P5	Sbt	32.28 ± 0.77
P5	Sat	0.00 ± 0.00
P6	Abt	31.38 ± 0.81
P6	Aat	31.91 ± 1.53
P6	Sbt	24.79 ± 0.16
P6	Sat	25.41 ± 0.59

Table S3 | Ct Measurements for Patients P1–P6. Values are reported as: $C_t \pm$ SD. Abbreviations: A = air, S = surface, bt = before treatment, and at = after H_2O_2 nebulisation. Undetectable signals are recorded as 0.

5 Mathematical Modelling of H_2O_2 Nebulisation Kinetics

We represent the decontamination dynamics induced by H_2O_2 nebulisation as a two-phase, piecewise process that separates (i) an initial *stabilisation* interval—capturing aerosol settling, instrument initiation and early room-specific transients—from (ii) a subsequent *log-linear* decay phase where the inactivation dynamics are well approximated by a first-order law with constant hazard. This choice provides an operational description that is both parsimonious and interpretable for short, sparsely sampled environmental time series, avoiding over-parameterised alternatives whilst retaining explicit control of the onset of effective decontamination.

Governing equation

Let t denote time (hours, h). The delay (onset) parameter is τ (h). For $t < \tau$, the effective virucidal action on the measured RNA load is negligible at the time scale of observation and the process is treated as stationary. For $t \geq \tau$, the decay of the environmental viral load is assumed first order with constant rate k (h^{-1}). Denoting by $N(t)$ the (possibly normalised) RT-qPCR load at time t and by N_0 the stabilised pre-onset level, the model reads

$$N(t) = \begin{cases} N_0, & t < \tau, \\ N_0 \exp\left[-k\,(t-\tau)\right], & t \geq \tau. \end{cases}$$

Equivalently, the post-onset dynamics satisfy the ODE

$$\frac{\mathrm{d}N}{\mathrm{d}t} = -k\,N, \qquad N(\tau) = N_0,$$

whose closed-form solution is classical in the theory of ordinary differential equations [5]. The characteristic *half-life* within the exponential phase is $t_{1/2} = \ln(2)/k$ (h), and the time to reach a fraction $\alpha \in (0,1)$ of N_0 after onset is $\tau + t_\alpha$ with $t_\alpha = \ln(1/\alpha)/k$.

Units and measurement conventions

All times (t and τ) are expressed in hours (h); the rate constant k is expressed in h^{-1}. In main-text figures, the vertical axis is labelled *Load (N)*. Unless explicitly stated otherwise, $N(t)$ denotes a normalised RNA-load proxy consistent with the RT-qPCR pipeline; when absolute units are used (e.g., copies mL^{-1} or per swab), they are indicated in the corresponding caption. For linear descriptive comparisons shown in Fig. 2, panel legends report β_1 (LS-Fit) for the experimental least-squares slope and β_1 (L-Fit) for the linear slope fitted to GAN-synthetic points, both expressed in units of load per hour ($\mathrm{N\,h^{-1}}$). GAN-synthetic points are used strictly to provide visual interpolation across sampling gaps and are not employed for inferential fitting.

Parameter estimation

Parameters (N_0, τ, k) are estimated by non-linear least squares (NLLS) on the piecewise model, with weights and error structure consistent with the experimental pipeline (see Methods in the main text). Because τ enters the model through a change of regime, we evaluate the objective over admissible grids of τ to ensure stable identification given the small number of time points; conditional estimates of (N_0, k) are then obtained by closed-form or NLLS updates. When reported, descriptive linear least-squares fits (LS-Fit) are provided solely as a benchmark for visual comparison and not as competing mechanistic models.

Model adequacy and small-sample model selection

Goodness-of-fit summaries (e.g., R^2) are reported as descriptive diagnostics but are *not* used to adjudicate among non-nested, non-linear specifications, since R^2 can be inflated under misspecification and is not an information criterion [6]. Formal selection balances fit and parsimony via the small-sample corrected Akaike Information Criterion (AICc), which is appropriate for the short series considered here (typically $n \approx 4$–5 observations per curve). This practice aligns with recent data-driven modelling in clinical-operational contexts under limited sample regimes, where information criteria and conservative uncertainty reporting are preferable to heuristic R^2 comparisons [7]. Evidence ratios and AICc weights (w_i) are computed in the standard manner from ΔAICc_i.

Interpretation and limitations

Within this piecewise framework, the delay parameter τ represents the emergent onset of effective decontamination at the environmental scale of observation, and should be read mechanistically as a coarse descriptor of room- and surface-level heterogeneities (e.g., aerosol settling, penetration and shielding) rather than as a microscopic latent period. The post-onset rate k aggregates multi-factor influences (agent concentration history, air exchange, surface properties) into a single hazard at the time scale resolved by the sampling protocol. With short series, τ can be weakly identified in regimes with shallow trends; in those cases, model choice is explicitly grounded on AICc. Uncertainty bands in figures reflect the error model described in Methods; all numerical summaries are accompanied by point estimates and intervals consistent with that specification.

Reproducibility notes

All code paths used to reproduce the piecewise fits, AICc tables and figure overlays are provided alongside the data. Figure-level colours and line styles (LS-Fit vs NLLS piecewise fits) match those in the main text to ensure one-to-one traceability from Methods to Supplementary Information.

References

[1] N. van Doremalen, T. Bushmaker, D. H. Morris, M. G. Holbrook, A. Gamble, B. N. Williamson, et al. "Aerosol and surface stability of SARS-CoV-2 as compared with SARS-CoV-1". *New England Journal of Medicine* 382.16 (2020), pp. 1564–1567. 10.1056/NEJMc2004973.

[2] A. W. Chin, J. T. Chu, M. R. Perera, K. P. Hui, H.-L. Yen, M. C. Chan, et al. "Stability of SARS-CoV-2 in different environmental conditions". *The Lancet Microbe* 1.1 (2020), e10. 10.1016/S2666-5247(20)30003-3.

[3] A. C. Walls, Y.-J. Park, M. A. Tortorici, A. Wall, A. T. McGuire, and D. Veesler. "Structure, Function, and Antigenicity of the SARS-CoV-2 Spike Glycoprotein". *Cell* 181.2 (2020). Accessed: 26 February 2025, 281–292.e6. 10.1016/j.cell.2020.02.058.

[4] D. Wrapp, N. Wang, K. S. Corbett, J. A. Goldsmith, C.-L. Hsieh, O. Abiona, et al. "Cryo-EM structure of the 2019-nCoV spike in the prefusion conformation". *Science* 367.6483 (2020), pp. 1260–1263. 10.1126/science.abb2507.

[5] A. Coddington and N. Levinson. *Theory of Ordinary Differential Equations*. International series in pure and applied mathematics. R.E. Krieger, 1984.

[6] A.-N. Spiess and N. Neumeyer. "An evaluation of R^2 as an inadequate measure for nonlinear models in pharmacological and biochemical research: a Monte Carlo approach". *BMC Pharmacology* 10 (2010). Tier 2 - Statistical critique justifying AIC over R^2, p. 6. 10.1186/1471-2210-10-6.

[7] A. C. Navas-Ortega, J. A. Sánchez-Martínez, P. García-Flores, C. Morales-García, and R. Fabregas. “Data-Driven Modelling of IRCU Patient Flow during the COVID-19 Pandemic”. *Computational and Structural Biotechnology Journal* 27 (2025), pp. 4657–4667. https://doi.org/10.1016/j.csbj.2025.10.017.
